# Online Short-Term Mindfulness-Based Intervention During COVID-19 Quarantine in Italy: Effects on Wellbeing, Stress, and Anxiety

**DOI:** 10.3389/fpsyg.2022.914183

**Published:** 2022-07-04

**Authors:** Teresa Fazia, Francesco Bubbico, Andrea Nova, Emilia Riggi, Giancarlo Caimi, Beril Calgan, Gerardo Salvato, Salvatore Bruno, Gabriella Bottini, Luisa Bernardinelli

**Affiliations:** ^1^Department of Brain and Behavioral Sciences, University of Pavia, Pavia, Italy; ^2^SSD Epidemiologia e Screening CPO, Universital Hospital “Città della Salute e della Scienza di Torino”, Torino, Italy; ^3^Cognitive Neuropsychology Centre, ASST “Grande Ospedale Metropolitano” Niguarda, Milan, Italy; ^4^Istituto di Psicosintesi, Milan, Italy

**Keywords:** mindfulness-based meditation, psychological wellbeing, COVID-19, lockdown, trait anxiety, state anxiety, stress

## Abstract

To limit the first spread of COVID-19 in March 2020, the Italian government imposed strict lockdown measures to the population. Despite necessary to reduce the virus transmission and the burden to the hospitals, social isolation has caused detrimental effects on psychological wellbeing and mental health. Moreover, during this period, it was also difficult to deliver psychological treatments and psychiatric assistance. A short (a weekly session for 9 weeks) mindfulness-based meditation program, named Integral Meditation (IM), was administered entirely online to healthy adults from Italy. This is a two-groups pre–post-quasi-experimental study in which the two groups, treated and control, were not randomized. Through matching procedures aimed at overcoming the absence of randomization, we analyzed a sample of 84 subjects (42 for each group). By applying linear mixed effect models, we tested the hypothesis of a beneficial effect of IM on wellbeing, perceived stress, and state anxiety, as measured by three self-reported questionnaires (WEMWBS, PSS, and STAI-X1, respectively), assuming that this effect could be different according to the level of baseline trait anxiety, as measured by STAI-X2. The results showed a statistically significant effect of STAI-X1 (β = −8.24 [95%CI −15.39; −1.09], *p* = 0.02) and WEMWBS (β = 4.61 [95%CI 0.94; 8.29], *p* = 0.01) in the higher trait anxiety subgroup only. No statistically significant effect of IM was observed for PSS. These results suggest that our IM, delivered online, may increase mental wellbeing and decrease anxiety specifically in subjects with higher trait anxiety.

## Introduction

On 31 December 2019, the health authorities of Wuhan identified the cause of the recent cases of “pneumonia of unknown cause,” found since October 2019 (World Health Organization). The strain responsible for these infections has been designated as severe acute respiratory syndrome coronavirus-2 (SARS-CoV-2), and the related disease was named, on 11 February 2020, by the World Health Organization (WHO) [World Health Organization Coronavirus disease (COVID-19) pandemic, 2019; World Health Organization Listings of WHO's response to COVID-19, 2020] as COVID-19 (https://www.who.int/). COVID-19 is an infectious disease belonging to the coronavirus family with dramatic multisystemic consequences primarily on the respiratory system (Su et al., 2016; Zhu et al., 2020). On 11 March 2020, WHO declared a global pandemic since the virus has spread rapidly in a growing number of countries.

To limit the number of infections and to protect people, various preventive measures have been promoted worldwide since March 2020 (Ministero della Salute) and each country has adopted different strategies regarding lockdown measures and daily life restriction (https://ig.ft.com/coronavirus-lockdowns/). At that time, Italy was one of the hardest-hit countries in the European Union and it adopted one of the most rigid lockdowns with not negligible social and psychophysical effects on the population. Every individual responded differently to this situation, also based on his/her socioeconomic status. Some of the possible detrimental effects generated by this situation, as also listed in the Istituto Superiore di Sanità website (https://www.iss.it/), are as follows: (*i*) the fear of losing livelihoods, not being able to work during isolation and getting fired; (*ii*) the feeling of helplessness, boredom, frustration, loneliness, and depression due to isolation; (*iii*) the possible anger and aggression against children, spouses, partners, and family members (increased family and intimate violence by the partner); (*iv*) the relapses of people with developing or existing mental health and substance use problems and other negative outcomes because they avoid health facilities or are unable to access their care providers; and (*v*) the worries about the future (Brooks et al., 2020; Giallonardo et al., 2020; Sepúlveda-Loyola et al., 2020).

In Italy, early researches regarding the effects of COVID-19 on the population have brought out alarming data, with 40–50% of adults reporting some sorts of psychological distress (Marazziti et al., 2020; Moccia et al., 2020; Favieri et al., 2021) and 30% of children and adults reporting high risk of developing post-traumatic stress disorder (PTSD) symptoms (Marazziti et al., 2020; Davico et al., 2021). These data are consistent with previous research on the Chinese population (Huang and Zhao, 2020; Sun et al., 2020), on the psychological consequences of social isolation in different scenarios (Brooks et al., 2020), and on the exposure to life-threatening situations on the human psyche (Nickell et al., 2004; Holmes et al., 2020; Roy et al., 2020). Furthermore, the fear caused by the possibility of contracting COVID-19, despite has been proved useful in motivating people to comply with the preventive rules issued by the governments, is considered a possible risk factor in the development of anxiety (Roy et al., 2020) and depression (Holmes et al., 2020). In addition, the sense of not having control over the situation and the inability to predict its evolution, both emblematic elements of the pandemic emergency, are substantial factors that characterize stress (Koolhaas et al., 2011). Chief among these, there was the difficulty or impossibility, given quarantine, for most of those who need psychiatric assistance to come into direct contact with mental health professionals. Psychological wellbeing of some categories, such as women and healthcare workers, was more affected by COVID-19. Healthcare workers were systematically exposed to emotional distress, anxiety, and sense of isolation that might represent a risk factor for later psychological issues, and these considerations are common within countries (Saladino et al., 2022). The risk of burnout was higher for frontline headline personnel working in intensive care units who were much more likely to be infected, which has caused an increased fear of infection (Lewis and Zauskova, 2021; Phillips and Kucera, 2021). Detrimental effects have been reported to be more pronounced for women, which reported more severe depression, distress symptoms (Coleman, 2020; Connor et al., 2020; Duncan, 2020; Lai et al., 2020; Vloo et al., 2021), and higher state and trait anxiety levels (Karasu et al., 2021), than for men.

Despite these figures present a daunting picture, there is an equally vast pool of research that offers simple, effective, and inexpensive solutions with which to circumvent the various limitations that the health emergency has entailed. In this regard, technological advancement has allowed to administer therapeutic psychoeducational and psychological interventions even remotely (Ho et al., 2020). The use of remotely delivered psychological therapies is not new in the field of telehealth, and several studies have documented its effectiveness, for example, in the treatment of patients with Parkinson's disease (Swalwell et al., 2018), anxiety disorders (Théberge-Lapointe et al., 2015; Berryhill et al., 2019), PTSD (Germain et al., 2009; Bolton and Dorstyn, 2015), panic disorders (Bouchard et al., 2004), and obsessive-compulsive disorder (Fitt and Rees, 2012).

In the early stage of the COVID-19 epidemic, many researchers highlighted the importance of providing interventions aimed at aid people's mental health (Duan and Zhu, 2020; Pfefferbaum and North, 2020). Particularly, in a study carried out in Italy (Giallonardo et al., 2020), the authors have underlined the future negative effects of the lockdown on the mental health among healthcare workers at the beginning of COVID-19 pandemic. Isolation, loneliness, and trauma were determined as the risk factors whereas coping strategies, resilience, internet use, social network, and post-traumatic growth were listed as protective factors. It has been emphasized the need for immediate efforts for developing preventive strategies as well as direct interventions aiming to mitigate the impact of the outbreak on individual and population mental health (Giallonardo et al., 2020). In this context, online intervention groups can become a place of social aggregation to support the mental health during COVID-19 lockdown, especially for those who experienced a period of loneliness, with all the negative effects that it entails.

Some authors have proposed the use of mindfulness-based interventions (MBIs), which incorporate the practice of meditation, to serve this purpose of aiding people's mental health (Baiano et al., 2020; Behan, 2020; Bursky et al., 2021; Green et al., 2021). The term mindfulness is strictly connected to the awareness, and it involves the intentional attention of the present moment experience, and the acceptance and non-judgement of such experience as it is, allowing thoughts to come and go without attachment and reaching a state of calm and relaxation (Chiesa et al., 2017; Behan, 2020). Online MBIs have already been deployed for years, and their effectiveness in the treatment of stress, anxiety, and in increasing the individual's general wellbeing, even after few weeks, is well recognized (Lunn et al., 2020).

In this regard, during the first total lockdown in Italy, Accoto et al. (2021) conducted an 8-week online mindfulness-based stress reduction (MBSR) protocol with 6 weeks of video support for meditation practice. The results showed an improvement in the capacity to choose not to react to negative thoughts instead of accepting their existence, and the treatment was found to be a protective factor against stress among the treated compared to the control group. Another study carried out in Italy during COVID-19 pandemic found a positive and protective value of the mindfulness practice on mindfulness, positive affect, depression, and insomnia (Bossi et al., 2022).

Our study is framed in this context, and it aims at evaluating the beneficial effect in improving wellbeing, stress, and state anxiety of a 9-week online MBI, named Integral Meditation (IM), administered for the first time online, on non-clinical people from the general population during the first COVID-19 lockdown in a country like Italy, where there has been one of the most rigid lockdowns having probably the widest implications. Our IM intervention first described in the study of Fazia et al. (2020a) was developed to promote personal and spiritual growth, especially within the general non-clinical population.

Specifically, this study aims to test the hypotheses that our intervention: (*i*) decreases state anxiety as measured by State Trait Anxiety Inventory (STAI-X1); (*ii*) decreases stress as measured by Perceived Stress Score (PSS); and (*iii*) increases wellbeing as measured by Warwick-Edinburgh Mental wellbeing Scales (WEMWBS) and that these effects were more pronounced in subjects' with higher baseline levels of trait anxiety, as measured by STAI-X2 questionnaire, which means in statistical terms to assume a three-way interaction between trait anxiety, time, and treatment. The choice to investigate these three endpoints (i.e., state anxiety, stress, and wellbeing) and formulate the above reported hypotheses was driven by the fact that these endpoints, worsen following the pandemic course (Brooks et al., 2020), and they are reported to mainly benefit from MBIs (Eberth and Sedlmeier, 2012; Khoury et al., 2015). Furthermore, we assumed that subjects who at the beginning of the study had higher levels of trait anxiety and thus had the tendency to respond to the concerns, troubles, and worries to various situations (Gidron, 2013; Taoka et al., 2014; Saviola et al., 2020; Fino et al., 2021), presumably were more affected by COVID-19 pandemic, and were more likely to benefit from our short-term online intervention than those having lower score. In addition, empirical studies suggest an inverse relationship between trait mindfulness and trait anxiety, so that people with higher trait anxiety are more likely to have a lower trait mindfulness (Jaiswal et al., 2019a). Being our intervention built to be easy-to-learn and to produce quick benefits also *via* an increase of mindfulness, there may be a higher possibility to detect a stronger observable beneficial effect among those people with a more urgent need for support and with low level of mindfulness.

## Materials and Methods

### Participants

We planned this research to offer to non-clinical Italian adults a quick but still complete mindfulness-based training, during the first wave of infection by COVID-19 and the relative confinement imposed by the Italian government, and to study its effect on three psychological endpoints. Therefore, during April 2020, we opened a call for an application to join the study promoted through digital advertising, i.e., mailing lists and social media posting. Given the nature of our intervention, participation was voluntary-based. Given the challenging time due to the pandemic, we felt unethical to randomize people to intervention and non-intervention. People who were interested in participating to our program and met the inclusion criteria were admitted and allocated to the treated group. While as to the control group, who did not receive any intervention, subjects interested in contributing to this research and meeting the inclusion criteria but, for various logistical issues unable to attend the classes, served as passive control group by only filling out the same questionnaires as the treated group. The inclusion criteria were as follows: (*i*) the absence of any psychiatric record, assessed by asking straight questions about these conditions. So, subjects suffering from severe anxiety or depression, severe mental illness (e.g., hypomania or psychotic episode), or any other serious mental or physical health problem were excluded from the study; (*ii*) to be 18 or older; (*iii*) to have a digital device and an Internet connection to complete the assessment; (*iv*) to install a dedicated app and to participate to videoconferences; and (*v*) to understand Italian language. To join the study, we asked the participants to provide agreement with the privacy and informed consent form that was sent to them by email. The study was not registered at Clinicaltrials.gov.

### Intervention

Our intervention consists of nine mindfulness-based meditation classes, given one time a week and lasting ≈60 min each on the Zoom videoconferencing platform (Zoom Video Communications, Inc. Global Infrastructure and Security Guide Global Infastructure and Security Guide) from April to July 2020. Our mindfulness-based IM program, well accepted by both novice and experienced meditators, has strong evidence of efficacy in the general population as reported in our previous studies (Fazia et al., 2020a,b, 2021) in which the same program was instead provided one time a week on 12 face-to-face sessions. A detailed description of the IM is reported in the study of Fazia et al. (2020a). Briefly, it incorporates mindfulness and aspects from different traditional meditation techniques albeit it shared many features with the classical mindfulness-based program (MBP) it differs to them especially in the use of the imagery to power the concentration. IM was slightly adapted here to meet the need of the target population in this unprecedent historical moment, for example by (a) reducing the number of sessions from twelve to nine, to guarantee a better adherence and participation albeit non-neglecting the main research objectives, and (b) by administering it online given the impossibility of carrying face-to-face classes. As for the duration, mindfulness programs should be lasted at least 8 weeks, even if Gotink et al. (2016) assess how an 8-week MBI induces neurofucntional changes similar to those observed after a longer practice. Being a total of nine online instead of 12 face-to face sessions of the original version, each cycle is not repeated for three meditation sessions in a row as in the study of Fazia et al. (2020a), but it is structured as follows:

Cycle 1: aware diaphragmatic breathing, keeping the posture (repeated for two meditation sessions in a row).
Cycle 2: body scan and awareness of body sensations (repeated for two meditation sessions in a row).
Cycle 3: emotions and thoughts feeling and releasing (repeated for two meditation sessions in a row).
Cycle 4: imagery activity to change the state of consciousness (repeated for three meditation sessions in a row).

In each cycle, also the abilities acquired in the preceding cycle/s are still practiced and new one is introduced, and, as a result, the abilities evolve across cycles. IM simultaneously uses breathing, focusing attention, release of physical tensions, thoughts, and feeling sensations through internal senses and imagery, allowing a quick relaxation and more deeply a physical, energetic, and spiritual wellbeing. At the beginning and the end of the meditation classes, the participants had the opportunity to socialize (even if remotely) and share their thoughts, feelings, and emotions with the other participants.

Here, we assessed its efficacy in helping to reduce and managing stress, reducing anxiety, increasing, and balancing psychological well-being by administering it for the first time online during an unprecedented situation such as the COVID-19 quarantine.

### Measures

Each participant, both in the treated and in the control groups, filled in three self-report psychological questionnaires, i.e., STAI-X1, PSS, and WEMWBS, at two different time points: at t0 (i.e., before the start of the study) and at t1 (i.e., at the end of the study). In addition, for each participant, we collected trait anxiety measures through STAI-X2 questionnaire, and sociodemographic information through a background questionnaire, at t0 only. Some information related to the impact of COVID-19 on participants' life was also collected at both t0 and t1, and the answers given at t0 are compared with those at t1 to obtain seven variables describing the impact of COVID-19 on participant's life during the studied time period. The seven COVID-related variables were used as covariates in the statistical analysis and provide information about the following: (i) if participants or (ii) someone in their inner circle (e.g., family, friends) or (iii) acquaintances have contracted the virus; (iv) if someone participants' inner circle or (v) acquaintances have died due to the virus; (vi) if participants have changed the people they lived with; and (vii) if participants have lost their job.

The participants completed the questionnaires online *via* Google Forms.

In detail, the psychological scales used were as follows:

STAI-X1 and STAI-X2 (State Trait Anxiety Inventory). The STAI questionnaire (Spielberger, 1970) is composed of two parts: the X1-scale assesses anxiety as a state to find out how the patient was feeling at the time of the assessment whereas the X2-scale assesses anxiety as a trait that reflects how a person generally feels. Each of them consists of 20 items, and responses are given in 4-point Likert scale. Participants are asked to classify themselves by given statements in the STAI-X1 scale as “not at all” (1) to “very much so” (4), while in the STAI-X2 scale as: “almost never” (1) to “almost always” (4). The values obtained in each of the scales range from 20 to 80 points, with the 20–40 range described as a low level of anxiety, 41–60 as moderate anxiety, and 61–80 as a high anxiety. We employed STAI-X1 as a dependent variable to measure the effect of the treatment on it. STAI-X2 was used to measure the baseline level of trait–anxiety before the intervention only and was used in the subsequent analysis as an explanatory interactor variable to test the hypothesis that the effect of treatment was different based on its value. The questionnaires have good psychometric properties in the English and Italian versions (Spielberger et al., 1983).

Perceived Stress Scale (PSS) (Cohen et al., 1983) is a 10-items questionnaire that measures the perception of stress and the degree to which situations in one's life are appraised as stressful during the last month. Answers were given on a 5-point scale and items were designed to tap how unpredictable, uncontrollable, and overloaded respondents find their lives. Higher scores are associated with a greater stress perception. The PSS has good psychometric properties in the English and Italian versions (Mondo et al., 2019). PSS scores were used as a dependent variable to evaluate the effect of the treatment on them.

Warwick-Edinburgh Mental Wellbeing Scale (WEMWBS) (Tennant et al., 2007) is a 14-items questionnaire of mental wellbeing including subjective wellbeing and psychological functioning. All items in a 5-point scale are worded positively and addressed the aspects of positive mental health and measure the frequency of the subject's attitudes from “never” to “always.” Higher scores indicate mental wellbeing. This questionnaire has good psychometric properties valid also in its Italian version (Gremigni and Stewart-Brown, 2011). WEMWBS scores were used as a dependent variable to evaluate the effect of the treatment on them.

### Statistical Analysis

This is a two-group pre–post-quasi-experimental design, in which participants were not randomly assigned to the treatment or to the control group. So to reduce confounding bias due to the absence of randomization, we adopted a nearest neighbor matching on the propensity score (PS), using the matchit R function with method nearest, implemented in MatchIt R package (Ho et al., 2011). Conditionally on the PS, using the match.data R function, we matched the treated and control subjects with 1:1 ratio with respect to putative measured confounding variables included in the PS (i.e., nationality, marital status, number of children, dependent children/family members, unpaid loans, years of education, job, and type of employment agreement). Using this procedure, the control group will have nearly the same distribution of the included variables as the treated one, thus eliminating differences between the two groups and increasing comparability, thereby correcting for selection bias (Graham, 2010). To avoid a severe reduction in the sample size due to variables which are poorly represented in the whole sample, not all the collected background variables were included in the PS calculation. The variables that we did not included in the PS calculation, which resulted to be stastically significant different among the two groups, were added as covariates in the models to estimate the treatment effect adjusting and controlling for residual confounding. All the models were also corrected for sex, age, and location during the study period as well as for the seven COVID-19-related variables. So, despite the absence of randomization, in this way, we are confident to have controlled for all possible measured confounders.

On the matched sample (numbers reported in the RESULTS section), baseline differences in all the sociodemographic and COVID-19-related variables between the two groups were investigated using Wilcoxon rank-sum test for non-normally distributed age and chi-squared test for all the categorical variables.

Each outcome was collected at two time points (i.e., t0 and t1) and subjects who did not fill in the proposed questionnaires were excluded from the analysis. Questionnaires were scored following the provided guidelines and for each questionnaire, internal consistency was assessed *via* Cronbach's α coefficient (Cicchetti, 1994). Differences between groups at baseline were investigated for each outcome using non-parametric Wilcoxon rank-sum test.

Data were analyzed following an intention to treat approach, i.e., independent of the number of classes completed. This approach allows having a conservative estimation of the treatment effect and avoiding biases related to dropouts and participants' adherence.

For the purpose of the analysis, STAI-X2 was further categorized into two categories as above or below the median, chosen as cutoff value. Linear mixed model effects (LME) (Pinheiro and Bates) have been applied to test the hypothesis of a beneficial effect of our intervention on the investigated outcomes. A random intercept for subjects in the form of 1|subject had been used to adjust the models for intra-subject variability produced by the two repeated measurements at t0 and t1 carried out on the same subject. Since we hypothesized a different effect of treatment based on higher or lower baseline trait anxieiy, we first tested the statistically significance of the coefficient of the three-way interaction between time, treatment and the categorized STAI-X2 score (i.e., time ^*^ treatment ^*^ categorized STAI-X2). If the three-way interaction was statistically significant, which means that the effect of treatment is different in the two groups of baseline trait anxieiy, we further estimated the effect of the interaction between time and treatment (i.e., time ^*^ treatment) separately in the two groups of trait anxiety (within-group pre–post-treatment differences). Instead, if the three-way interaction was not statistically significant, we estimated the effect of the interaction between time and treatment in the whole sample without stratification. The coefficient of the interaction time ^*^ treatment measures the difference in slopes between the two treatment groups, indicating how much more the treatment group is improving over time with respect to the investigated endpoints, compared to the control group over the same period. Normality of residuals was assessed graphically through Q-Q plots and Shapiro–Wilk test.

In addition, for testing the possible dose–response effect of each endpoint in the treated group, a linear model was fitted by specifying as dependent variable, the post-intervention questionnaire's score, and as predictors, the number of classes attended by each participant, the baseline questionnaire's measure, sex, and age.

*p*-Value ≤ 0.05 was considered as statistically significant. Given the explanatory nature of our study, limited number of hypothesis tested (*n* = 3), and the need to avoid missing important findings, no multiple testing correction was applied (Rothman, 1990).

Descriptive statistics are reported for both groups as the means ± standard deviation (SD). All analyses were done using R 3.5.1 software (R Core Team).

## Results

**Figure 1:**
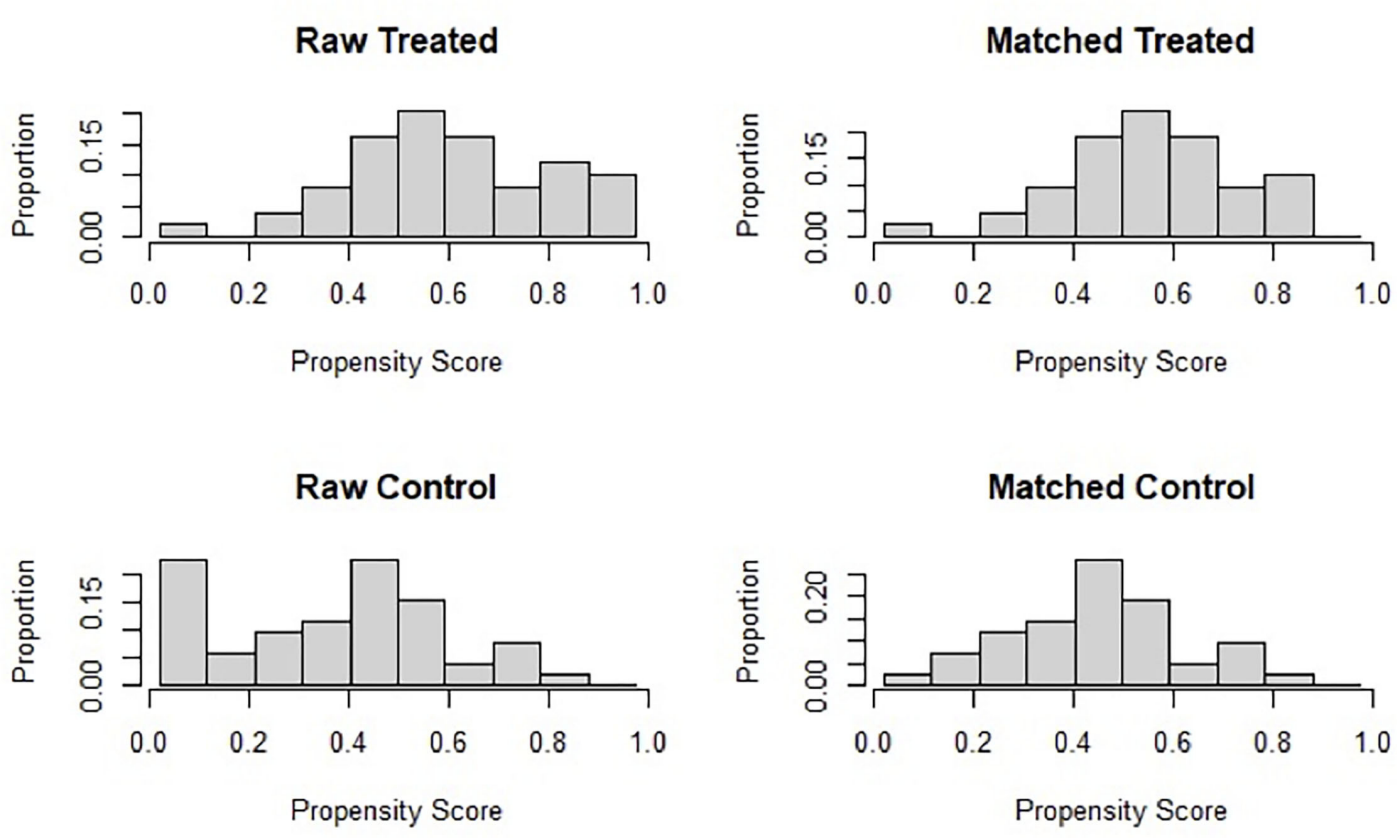
Participants' flow diagram.

**Figure 2:**
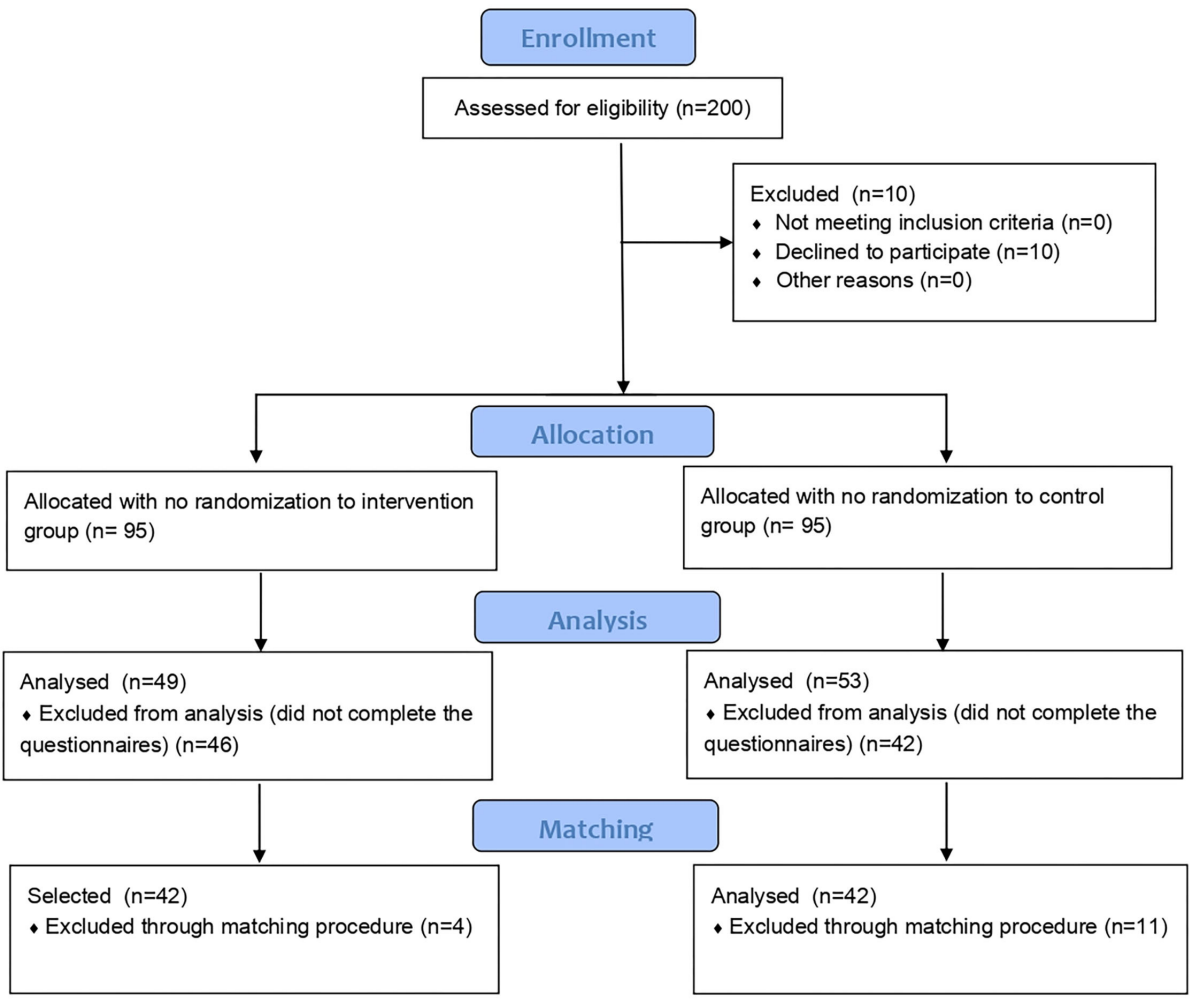
Histogram of the density of propensity score distribution in the two groups, i.e., treated and control, before (Raw) and after the matching procedure (Matched).

During the recruitment phase, 95 voluntary participants were enrolled in the treated group, and simultaneously, the same numbers of participants were recruited in the control group. Among them, we excluded from the analysis those who did not fill in the post-treatment questionnaires, so that 102 participants (treated = 49, control = 53) were eligible for the study. Through the matching procedures, a final sample of 84 subjects (treated = 42, control = 42) were selected for the analysis. The sample consisted of 62 females and 22 males with a mean age±(SD) of 45.33 ± (15.04) ranging from 18 to 75. Participants' flow diagram is represented in Figure 1. To evaluate the effectiveness of the matching procedure, in Figure 2 the histogram of the density of PS distribution in the two groups before and after the matching procedure is plotted. Before matching (raw), treated groups have a different distribuition of PS than the control group; after matching, the density distributions of the two groups become somewhat more similar.

The sample size was determined by the feasibility of recruitment. For the analyzed sample size (*n* = 84 subjects) and for an expected medium effect size *d* = 0.50, power analysis was determined *post-hoc* and was equal to 0.80 with the alpha error equal to 0.05.

Study sample characteristics, including the COVID-related variables, are provided in Table 1. No differences were observed between the two groups at baseline characteristics except for the variables sport (*p* = 0.005), time spent on sport activities (*p* = 0.01), and previous meditation experience (*p* < 0.0001) as well as for having acquaintances who died for COVID-19 during the period of the study (*p* = 0.05) as illustrated in Table 1. These variables were included as covariates in the LME models to adjust the estimate of interest for confounding. Models were also adjusted for sex, age, and all the seven COVID-related variables, being variables not included in the matching procedure by PS.

**Table 1:** Baseline characteristics of the analyzed sample (treated = 42, controls = 42).

**Variables**	**Mean (SD) controls**	**Mean (SD) treated**	***p*-value^**a**^**
**Age**	48.14 (15.33)	42.52 (14.37)	0.08
	***N*** **(%) controls**	***N*** **(%) treated**	
**Sex**			
Male	14 (33%)	8 (19%)	0.21
Female	28 (67%)	34 (81%)	
**Nationality**			
Italian	41 (98%)	41 (98%)	1
Non-Italian	1 (2%)	1 (2%)	
**Marital status**			
Cohabitant/married	24 (57%)	27 (64%)	0.78
Unmarried/single	16 (38%)	13 (31%)	
Separated/Divorced	2 (5%)	2 (5%)	
**Number of children**			
0	19 (45%)	16 (38%)	0.58
1	6 (14%)	11 (26%)	
2	14 (33%)	13 (31%)	
≥3	3 (7%)	2 (5%)	
**Dependent children/family members**			
No	27 (64%)	29 (69%)	0.82
Yes	15 (36%)	13 (31%)	
**Unpaid loans**			
No	34 (81%)	38 (90%)	0.35
Yes	8 (19%)	4 (10%)	
**Education**			
Middle school	3 (7%)	3 (7%)	0.72
High school	21 (50%)	16 (38%)	
Degree	15 (36%)	20 (48%)	
Post-graduate course (e.g., PhD)	3 (7%)	3 (7%)	
**Job**			
Employed	28 (67%)	27 (64%)	1
Not-employed	14 (33%)	15 (36%)	
**Type of employment agreement**			
Undetermined term	2 (5%)	1 2(%)	0.72
Fixed term	20 (48%)	23 (55%)	
Not applicable	20 (48%)	18 (43%)	
**Sport**			
No	16 (38%)	4 (10%)	**0.005**
Yes	26 (62%)	38 (90%)	
**Time spent on sport activities**			
Every day	5 (12%)	6 (14%)	
Three times a week	8 (19%)	10 (24%)	0.01
Once/twice a week	10 (24%)	20 (48%)	
Never/Rarely	19 (45%)	6 (14%)	
**Smoker**			
No	33 (79%)	38 (90%)	0.23
Yes	9 (21%)	4 (10%)	
**Previous meditation experience**			
No	32 (76%)	5 (12%)	**<0.0001**
Yes	10 (24%)	37 (88%)	
**Religious**			
No	18 (43%)	22 (52%)	0.51
Yes	24 (57%)	20 (48%)	
**Member of a cultural/sportive association**			
No	30 (71%)	28 (67%)	0.81
Yes	12 (29%)	14 (33%)	
**You contracted COVID-19 during the study period**			
No	36 (86%)	37 (88%)	1
Yes/maybe	6 (14%)	5 (12%)	
**Someone among family or friends contracted COVID-19 during the study period**			
No	13 (31%)	16 (38%)	0.72
Maybe	7 (17%)	5 (12%)	
Yes	22 (52%)	21 (50%)	
**Acquaintance(s) contracted COVID-19 during the study period**			
No	10 (24%)	4 (9%)	0.09
Maybe	6 (14%)	3 (7%)	
Yes	26 (62%)	35 (83%)	
**Someone among family or friends died for COVID 19 during the study period**			
No	36 (86%)	33 (79%)	0.57
Yes	6 (14%)	9 (21%)	
**Acquaintance(s) died for COVID-19 during the study period**			
No	24 (57%)	14 (33%)	**0.05**
Yes	18 (43%)	28 (67%)	
**Lost job during the study period**			
No	36 (87%)	32 (76%)	0.4
Yes	6 (13%)	10 (24%)	
**Location during the study period**			
Central Italy/South Italy	9 (21%)	4 (9%)	0.32
North Italy	31 (74%)	36 (85%)	
Out of Italy	2 (5%)	2 (5%)	
**Change in cohabitation during the study period**			
No	36 (87%)	33 (79%)	0.57
Yes	6 (13%)	9 (21%)	

^a^*p-value for between groups comparison*.

*p-values ≤ 0.05 are considered as statistically significant and highlighted in bold*.

Mean, SD, and internal consistency for each questionnaire at both t0 and t1 are reported separately in the control and in the treated groups in Table 2. In Table 2, the *p*-values of baseline differences for each questionnaire were also reported and no statistically significant differences were observed except for STAI-X2 that was used as an interactor in the model and so do not represent an endpoint.

**Table 2:** Mean, Standard Deviation (SD) and internal consistency for each questionnaire and subscale in the two groups (controls and treated) at both time points (to and t1).

**Questionnaire**	**Mean (SD) controls t0**	**Mean (SD) treated t0**	***p*-value**	**Mean (SD) controls t1**	**Mean (SD) treated t1**	**Internal consistency t0**	**Internal consistency t1**
*STAI-X1*	41.31 (9.19)	42.86 (10.68)	0.48	41.83 (10.76)	40.52 (11.18)	0.94	0.96
*STAI-X2*	39.64 (9.75)	44.24 (9.66)	0.03	–	–	0.91	–
*PSS*	16.55 (7.74)	17.45 (6.92)	0.57	15.12 (7.13)	15.69 (6.46)	0.89	0.91
*WEMWBS*	50.12 (8.38)	48.14 (7.9)	0.71	48.50 (10.04)	49.29 (8.36)	0.91	0.94

*P-values of the baseline differences between group is also reported*.

*SD, Standard Deviation; –:questionnaire collected at t_0_ only*.

The continuous STAI-X2 score ranging from 22 to 63 was dichotomized using as cutoff its median value (=42). Thus, the sample was split into two categories: lower STAI-X2 with values ≤ 42 and higher STAI-X2 with values >42. A total of 43 subjects (24 controls and 19 treated) were assigned to the group with lower STAI-X2 whereas 41 subjects (18 controls and 23 treated) were assigned to the group with higher STAI-X2.

By testing the three-way interaction (time ^*^ treatment ^*^ categorized STAI-X2), we investigated whether the effect of our IM was different in the two groups (treated and control) at the two different levels of trait anxiety. A statistically significant three-way interaction, which means that there is a different effect of treatment in the two groups of trait anxiety, was found for STAI-X1 (β = −10.08 [95%CI −18.46; −1.70], *p* = 0.019) as well as for WEMWBS (β = 4.76 [95%CI 0.01; 9.50], *p* = 0.049) while not in PSS (β = 6.01 [96%CI −2.36; 14.38], *p* = 0.157). Full results are reported in Table 3.

**Table 3:** Results of the linear mixed models for the effect of treatment and categorized STAI-X2 on the investigated outcomes.

**Questionnaire**	**Predictors**	**β [95%CI]**	***p*-value**
**STAI X-1**			
	(Intercept)	39.01 [29.35; 48.67]	**<0.001**
	Treatment (treated)	3.39 [−4.32; 11.09]	0.383
	Time (t1)	−2.05 [−5.91; 1.81]	0.294
	Categorized STAI-X2 (High)	6.38 [−0.35; 13.11]	0.063
	Sex (M)	−1.23 [−6.09; 3.63]	0.616
	Age	−0.02 [−0.17; 0.12]	0.731
	Previous meditation experience (yes)	−4.72 [−10.15; 0.70]	0.087
	How often sport	−1.31 [−3.66; 1.04]	0.270
	Change cohabitation (yes)	−3.37 [−8.52; 1.79]	0.197
	contracted COVID-19 (yes/maybe)	−0.46 [−6.62; 5.71]	0.883
	Friends/family contracted COVID-19 (maybe)	4.33 [−2.65; 11.30]	0.220
	Friends/family contracted COVID-19 (yes)	0.74 [−4.01; 5.48]	0.758
	Acquaintance contracted COVID-19 (maybe)	−0.99 [−9.17; 7.20]	0.811
	Acquaintance contracted COVID-19 (yes)	4.82 [−1.41; 11.05]	0.127
	Friends/family bereavement (yes)	3.85 [−1.54; 9.23]	0.159
	Acquaintance bereavement (yes)	−2.32 [−7.13; 2.49]	0.339
	Job lost (yes)	2.38 [−3.00; 7.75]	0.381
	Location (out of Italy)	0.12 [−10.89; 11.13]	0.983
	Location (nord)	0.15 [−4.90; 5.20]	0.953
	Time * treatment	1.94 [−3.86; 7.75]	0.507
	Treatment * categorized STAI-X2	0.57 [−8.66; 9.80]	0.902
	Time * categorized STAI-X2	6.01 [0.04; 11.97]	**0.048**
	Time * treatment * categorized STAI-X2	−10.08 [−18.46; −1.70]	**0.019**
**PSS**			
	(Intercept)	14.72 [8.95; 20.49]	**<0.001**
	Treatment (treated)	5.46 [0.46; 10.45]	**0.033**
	Time (t1)	1.11 [−2.76; 4.99]	0.569
	Categorized STAI-X2 (High)	5.08 [0.55; 9.62]	**0.029**
	Sex (M)	2.60 [−0.17; 5.37]	0.065
	Age	−0.00 [−0.08; 0.08]	0.957
	Previous meditation experience (yes)	−2.74 [5.81; 0.34]	0.080
	How often sport	−1.14 [−2.47; 0.20]	0.094
	Change cohabitation (yes)	−3.34 [−6.27; −0.42]	**0.026**
	contracted COVID-19 (yes/maybe)	−3.50 [−7.00; 0.01]	0.051
	Friends/family contracted COVID-19 (maybe)	6.29 [2.29; 10.30]	**0.003**
	Friends/family contracted COVID-19 (yes)	2.60 [−0.10; 5.29]	0.059
	Acquaintance contracted COVID-19 (maybe)	−5.09 [−9.75; −0.43]	**0.033**
	Acquaintance contracted COVID-19 (yes)	0.21 [−3.32; 3.74]	0.906
	Friends/family bereavement (yes)	1.27 [−1.78; 4.33]	0.408
	Acquaintance bereavement (yes)	−1.67 [−4.40; 1.06]	0.226
	Job lost (yes)	1.69 [−1.36; 4.74]	0.272
	Location (out of Italy)	−2.46 [−9.29; 4.37]	0.475
	Location (nord)	0.28 [−2.88; 3.44]	0.859
	Time * treatment	−2.85 [−8.68; 2.98]	0.333
	Treatment * categorized STAI-X2	−4.26 [−10.53; 2.01]	0.180
	Time * categorized STAI-X2	−6.07 [−12.01; −0.12]	**0.046**
	Time * treatment * categorized STAI-X2	6.01 [−2.36; 14.38]	0.157
**WEMWBS**			
	(Intercept)	54.97 [48.00; 61.94]	**<0.001**
	Treatment (treated)	−2.64 [−8.12; 2.84]	0.340
	Time (t1)	−0.62 [−2.80; 1.55]	0.570
	Categorized STAI-X2 (High)	−10.66 [−15.40; −5.92]	**<0.001**
	Sex (M)	−1.48 [−5.03; 2.08]	0.410
	Age	0.02 [−0.08; 0.13]	0.663
	Previous meditation experience (yes)	1.82 [−2.15; 5.79]	0.364
	How often sport	1.61 [−0.11; 3.33]	0.067
	Change cohabitation (yes)	1.91 [−1.87; 5.69]	0.316
	contracted COVID-19 (yes/maybe)	−1.08 [−5.59; 3.44]	0.636
	Friends/family contracted COVID-19 (maybe)	0.34 [−4.73; 5.42]	0.894
	Friends/family contracted COVID-19 (yes)	1.35 [−2.13; 4.83]	0.442
	Acquaintance contracted COVID-19 (maybe)	0.28 [−5.71; 6.27]	0.925
	Acquaintance contracted COVID-19 (yes)	−4.35 [−8.93; 0.22]	0.062
	Friends/family bereavement (yes)	−3.24 [−7.19; 0.71]	0.106
	Acquaintance bereavement (yes)	1.49 [−2.04; 5.02]	0.403
	Job lost (yes)	−2.75 [−6.69; 1.20]	0.169
	Location (out of Italy)	−0.30 [−7.77; 7.17]	0.936
	Location (nord)	−1.02 [−4.42; 2.39]	0.553
	Time * treatment	0.47 [−2.81; 3.74]	0.777
	Treatment * categorized STAI-X2	1.60 [−4.88; 8.07]	0.625
	Time * categorized STAI-X2	−2.34 [−5.72; 1.04]	0.173
	Time * treatment * categorized STAI-X2	4.76 [0.01; 9.50]	**0.049**

*For each questionnaire β coefficients, their 95% CI, and p-values are reported*.

*p-values ≤ 0.05 are considered as statistically significant and highlighted in bold*.

When statistically significant three-way interaction was observed, that is for STAI-X1 and WEMWBS, a further analysis on these two endpoints was performed stratifying the data according to the trait anxiety groups and testing the effect of time ^*^ treatment separately within each group. No statistically significant results were found in the group with lower baseline value of STAI-X2 for both STAI-X1 and WEMWBS. While in the group with higher STAI-X2, a statistically significant time ^*^ treatment interaction was observed both in STAI-X1 (β = −8.24 [95%CI −15.39; −1.09], *p* = 0.02) and in WEMWBS (β = 4.61[95%CI 0.94; 8.29], *p* = 0.01). These latter results support the hypothesis of a beneficial effect of our intervention on decreasing state anxiety and increasing wellbeing in people having higher levels of baseline trait anxiety.

With regard to PSS in which no statistically significant three-way interaction was observed, a LME model on the whole sample was fitted for testing the interaction between time and treatment but no statistically significant difference between pre–post-changes in the two groups was found (β = −0.29 [95%CI −4.46; 3.87], *p* = 0.889), thus indicating that our intervention was not effecting in managing and reducing stress.

As for the number of IM session attendend, among the 42 treated subjects, 14 (33%) attended more than seven meditation classes, and 25 (60%) attended a number of classes between three and seven, whereas 3 (7%) attended <2 classes, with 5.43 mean number of classes attended. We did not find any statistically significant effect of the number of attended meditation classes on the investigated endpoint, Specifically, the estimate and the *p*-value of the effect of the number of attended meditation classes as from the fitted linear model were as follows: β = −0.96 with *p* = 0.22 for STAI-X1, β = −0.69 with *p* = 0.19 for PSS and, β = 0.56 with *p* = 0.22 for WEMWBS, which means that no dose–response relationship with the number of meditation classes was observed in our study.

## Discussion

The aim of this study was to investigate the efficacy of our IM program on improving stress, state anxiety, and wellbeing as measured by three self-reported questionnaires, i.e., PSS STAI-X1, and WEMWBS, respectively. Our IM may be classified as a MBP since it was created by adopting MBP fundamental parts and the variable features chosen according to the characteristics of our target population, i.e., healthy adults from Italy. This study took place during the first lockdown period during the COVID-19 pandemic in Italy; therefore, the intervention was administered *via* an online platform. The study has good ecological validity since it involved the participants while they were at their home and facing a new situation for a period of 9 weeks. Our intervention was aimed at providing them a tool to deal with the negative effects induced by the coronavirus pandemic on psychological health.

Being trait anxiety, as measured through STAI-X2 questionnaire, part of an individual's personality (Spielberger, 1972) and related to a tendency to respond with concerns, troubles, and worries to various situations (Saviola et al., 2020), we hypothesized that people with baseline higher levels of STAI-X2 scores were more likely to benefit from our short-term online intervention than those having lower scores. To verify this hypothesis, we considered the median-based categorization of the STAI-X2 scores (lower vs. high) to act as an interactor, thus modifying the effect of treatment. Statistically significant interaction implies that in a particular group, the effect of treatment could be more marked than in the other, and thus, analyzing the global sample, without considering this, a dilution of the treatment effect masking possible statistical significance could happen. In other words, people with lower levels of trait anxiety tend to have a better response to temporary stressful events (state anxiety); in contrast, individuals with higher levels of trait anxiety tend to have a worsen response to temporary stressful events (state anxiety) and could have a greater beneficial effect of our intervention. Furthermore, a growing body of evidence from literature has demonstrated the inverse relationship between mindfulness and trait anxiety: individuals with a higher predisposition to mindfulness will be those with lower levels of trait anxiety (Arch and Craske, 2010; Mankus et al., 2013; Jaiswal et al., 2018, 2019a,b). Interestingly, such inverse relationship also influences the cognitive functioning. For instance, Jaiswal et al. (2018) grouped participants based on their mindfulness and trait anxiety scores showing that the group with high mindfulness and low anxiety had higher efficiency of cognitive control. In a meta-analysis, it has been reported that the effect size of transcendental meditation technique on reducing trait anxiety depends on the patients' initial anxiety level (Orme-Johnson and Barnes, 2014). The principle that populations with elevated initial levels of an outcome will show larger effect sizes has been found also for other variables, such as depression and blood pressure. An EEG study also reported a differential effect of meditation based on participants' initial level of trait anxiety, suggesting that having lower trait anxiety more readily induces meditation with a predominance of an internalized attention while higher trait anxiety more readily induces meditation with a predominance of relaxation (Murata et al., 2004).

We found a statistically significant three-way interaction between time, group, and categorized STAI-X2, meaning that the effect of our intervention is different depending on having higher or lower levels of trait anxiety, both in STAI-X1 and in WEMWBS. In the subsequent stratified analysis, the results showed that in the group with higher trait anxiety, there was a statistically significant effect of treatment both in STAI-X1 and in WEMWBS. No statistically significant effect of treatment in the investigated outcomes was found in the group with lower STAI-X2 scores. The statistically significant results found in the high trait anxiety group but not in the low trait anxiety group confirm our hypothesis that a beneficial effect of our IM is more remarkable in this group of individuals. A possible explanation of these different effects could reside in the so-called well-known ceiling effect as it happens in the study of Matiz et al. (2020), where participants with low-resiliency obtained better results than those having higher resilience, since these latter had baseline healthy profiles. In our sample, the participants with higher trait anxiety vs. the participants with lower trait anxiety have higher baseline mean scores in PSS (18.46 vs. 15.60), STAI-X2 (46.29 vs. 38.07) and lower mean scores in WEMWBS (43.46 vs. 54.53). Another explanation could also be the probably higher motivation to attend mindfulness classes that may have subjects with psychological suffering as are those with higher trait anxiety in our study. Participants who are in a worse condition, in fact, could be more motivated to engage for their own recovery as described in other works (Hayes and Plumb, 2007; Matiz et al., 2018). As for PSS score, our intervention had no effect on this endpoint either globally or separately in the two groups of STAI-X2. This latter result is in contrast to our hypothesis about the beneficial effect of meditation on perceived stress during the quarantine period but even if not statistically significant, it is important to note that the direction of the effect observed in terms of reduction of stress, as also emerged from the CIs was that expected. The lack of significance may be probably due to the limited recruited sample size, further reduced due to the matching procedures necessary to face the lack of randomization.

During COVID-19 pandemic, research works were performed to investigate the effect of different MBI on psychological indicators of wellbeing in different target populations, even if, as far as we know, ours represent the first study performed during the first lockdown testing the hypotheses of a beneficial effect of a MBI on wellbeing, stress, and state anxiety different based on baseline levels of trait anxiety on general non-clinical population. For example, a cross-sectional study (Priyanka and Rasania, 2021) during COVID-19 pandemic on the general population reported higher mental wellbeing scores and lower risk of psychological distress and depression in subjects practicing yoga and meditation; The study of Nutting et al. (2022) conducted on family physicians showed a reduction of anxiety, stress, and an increasing in perceiving resilience and compassion after a brief mindfulness intervention. A positive effect of MBI was also observed in the study by González-García et al. (2021) where a brief online mindfulness and compassion-based intervention increase mental health on first-year psychology students from a university in Spain during COVID-19 pandemic, and in Desai et al. (2021) where an 8-week heartfulness meditation program showed a positive impact on stress and sleep quality.

To summarize, our intervention worked more efficiently on those people who tended to react in a more anxious manner to the events and therefore having more difficulties to cope with the stressful situations.

Our study suffers from some limitations: (i) the absence of monitoring of the number of meditations performed by the participants in their free time. Ideally, future research should add such measures, as the effect of meditation becomes more evident with frequency; (ii) the small sample size, which may be a possible cause for the non-significance of PSS; (iii) given the nature of our intervention, enrolment was obviously on voluntary basis and participants probably were people interested in the theme of meditation and willing to follow this practice, and this could represent only a problem of generalization, i.e., these results may be only applicable to people who are willing to meditate, but not a bias; (iv) given the difficult time due to pandemic, we felt unethical to randomize people to intervention and non-intervention, so this is a quasi-experimental study with its intrinsic limitation that was faced by adopting appropriate statistical method; (v) even if we used the PS to overcome possible non-comparability of the two treatment groups due to the absence of randomization, group differences could still be present due to on unmeasured variables, as it happens in all observational studies; (vi) furthermore, although we improved internal validity *via* covariate balancing, we have removed subjects with no match and in this way we could have introduced problem in generalizability; (vii) the control group was passive so not involved in any activity except to fulfill the questionnaires; for such reason, our study is not evaluating exclusively the effectiveness of the intervention, since the changes in the treated group can be promoted by the mere participation in a new activity; and (viii) finally, we did not collect information about use of medication and/or drug for decreasing stress and anxiety that could confound the effect of our treatment, but being a non-clinical population in consideration of the adopted inclusion criteria, we can assume the absence or at least limited use of drug with negligible effect.

Despite the above reported limitation, we contributed in some ways to understanding the emotional impact of the pandemic on the general non-clinical population living in Italy. The results obtained provide more insight into the understanding of the beneficial effect of our proposed free and easily accessible specific IM training, already tested in previous studies (Fazia et al., 2020a,b, 2021) to be effective for improving psychological wellbeing even in novice meditators. It should be noted that the intervention was administered online for the first time and investigated during the stressful and overwhelming period of COVID-19 quarantine.

## Conclusion

As of 21 September 2021, more than 228 million people were infected with COVID-19, and more than 4 million and half deaths have been reported globally (World Health Organization).

The restrictions imposed by the Italian government due to COVID-19 pandemic have taken a great toll. The number of risk factors for an individual's mental wellbeing is manifold; ranging from being confined to a limited space for an extended period, not having certainties about the future, the possibility of losing one's job and being unable to reach a social environment are just the few examples. In this scenario, the possibility to engage in an activity designed to provide the tools to best cope with such stressors can be of enormous benefit to many individuals. Our project aimed at testing the efficacy of a particular mindfulness-based intervention in offering such help. Additionally, given the impossibility of gathering individuals in a face-to-face group, the intervention should have been held up in a virtual setting, in this case using videoconference meeting technologies.

Despite the impossibility to come in direct contact with the meditation group, participants showed improved outcomes on anxiety and mental wellbeing measures, even after a relatively short period of 9-week meditation training, and this happened in the high trait anxiety group.

Such results open a great possibility regarding public mental health services, offering a viable alternative to those services precluded to many individuals in remote areas or in time such as those lived in many areas of the world during the quarantine period. Future study on this topic should be performed to assess the longer-term impacts of the intervention, such as whether people have continued to meditate and how this has influenced their wellbeing. Additionally, further studies should investigate the generalizability capacity of a similar intervention in organizational contexts, such as schools, hospitals, and industries.

## Data Availability Statement

The original contributions presented in the study are included in the article/Supplementary Material, further inquiries can be directed to the corresponding author/s.

## Ethics Statement

The study was approved by the Ethics Committee of Department of Brain and Behavioral Sciences (protocol code 49/2020 on 27 April 2020). The patients/participants provided their written informed consent to participate in this study.

## Author Contributions

Conceptualization: TF, SB, and LB. Data curation and writing—original draft: FB and TF. Formal analysis: FB, TF, AN, ER, and BC. Funding acquisition: LB. Methodology: TF and LB. Supervision: LB. Writing—reviewing and editing: TF, FB, AN, ER, GC, BC, GS, SB, GB, and LB. All authors have read and agreed to the published version of the manuscript. All authors contributed to the article and approved the submitted version.

## Funding

We wish to thank the crowdfunding platform of the University of Pavia Universitiamo for helping us in the funding acquisition.

## Conflict of Interest

The authors declare that the research was conducted in the absence of any commercial or financial relationships that could be construed as a potential conflict of interest.

## Publisher's Note

All claims expressed in this article are solely those of the authors and do not necessarily represent those of their affiliated organizations, or those of the publisher, the editors and the reviewers. Any product that may be evaluated in this article, or claim that may be made by its manufacturer, is not guaranteed or endorsed by the publisher.
